# The Use of the Microscope

**Published:** 1885-11

**Authors:** 


THE USE OF THE MICROSCOPE. By Dr. Carl Friedlander, Privat Docent in Pathological Anatomy at Berlin. Translated by Henry C. Coe, M. D. 8vo., pp. 189. D. Appleton & Co. New York. 1885.

This little work is designed for beginners in microscopy, and describes the microscope, accessory apparatus and the various reagents necessary for carrying on microscopical work. It further gives methods for the preparation and examination of living tissues, fluids and bacteria found in disease. There are many works on this subject, which have appeared from time to time, and some of them are more comprehensive and fuller for the student than the one at hand. Still, this one is practical in its nature, and contains many valuable suggestions. H. W.

